# Editorial: Periodontal Disease – A Public Health Problem

**DOI:** 10.3389/fpubh.2015.00278

**Published:** 2016-01-08

**Authors:** Alexandrina L. Dumitrescu

**Affiliations:** ^1^Dental Private Practice, Bucharest, Romania

**Keywords:** periodontitis, gingivitis, inflammation, public health, health literacy, health education

**The Editorial on the Research Topic**

**Periodontal Disease – A Public Health Problem**

In 2010, the World Health Organization published an important report entitled “Equity, Social Determinants and Public Health Programmes” (1, 2) revealing that among priority public health conditions, oral health represents a global problem linked to inequalities in social circumstances (3). Periodontal disease, a chronic inflammatory disease resulting in progressive attachment and alveolar bone loss, is, after dental caries, one of the most important oral diseases contributing to the global burden of chronic disease (4), and it meets the criteria for consideration as a public health problem that requires action, as defined by Thomson et al. (5): i) it is widespread as affects “more than 50% of the adult population, while its severe forms affect 11% of adults, making severe periodontitis the sixth most prevalent disease of mankind” (6); ii) periodontitis severely impairs the individuals’ oral health-related quality of life (7, 8), their self-esteem (9) and their general well-being (10). Furthermore, several research studies have associated periodontal disease with various systemic diseases and conditions, such as diabetes, HIV, atherosclerotic vascular disease, rheumatoid arthritis, adverse pregnancy outcomes, obesity, and metabolic syndrome (11–18); iii) the costs of treating the periodontal disease are substantial (19–22); and iv) due to the current state of knowledge of the risk factors implicated in the etiopathogenesis of periodontal disease [e.g., smoking (23, 24), alcohol (25–27), poor diet, lack of exercise (28–30), stress, distress, and psychological-coping resistance (31–34)], there is sufficient information to allow the effective control of the common forms of the disease.

Several strategies of controlling periodontal disease have been described, such as the population strategy that requires use of the public health approach for changing health behaviors (e.g., smoking, oral self-care) (35), the secondary prevention strategy, and a high-risk strategy in persons at special risk (5). As non-communicable chronic diseases share numerous risk factors with periodontal diseases (18), The Common Risk Factor Approach (CRFA) and the health promotion approach have been acknowledged as key aspects of the strategies aimed at those who are known to be at high risk (36, 37).

Within the dental profession globally, for years, the treatment, control, and prevention of periodontal disease has been under the dominance of the biomedical model emphasized on the molecular and biological basis of disease at the individual level and focused on a professional dependency representation with clinical surgical procedures and on-going maintenance therapy (38). The preventive component in this patient-centered approach was oriented on improving patient oral health and to perform chairside advice and counseling on smoking behaviors (39, 40). Several limitations of this traditional clinical approach have been revealed such as (a) the high costs and therefore the limited access for socially disadvantaged, disabled, chronically ill and old populations with high need (41) and (b) it has only immediate short-term positive changes in oral hygiene, bleeding indexes, and knowledge, being ineffective in achieving constant improvements in periodontal outcomes at a population level (42, 43).

Nowadays, a broader and more comprehensive approach is used in periodontal disease prevention with interventions targeting both local etiologic as well as general risk factors (36). Unfortunately, in both developing and developed countries, the human, financial, and material resources are still in short supply to meet the necessities of oral health care services and to provide universal access, especially in disadvantaged communities (44), the cost of providing dental treatment for periodontitis patients is high and comparable with some non-communicable diseases (20, 45, 46), while periodontal treatment is scarcely included in oral health-care reimbursement schemes, if available (3). Furthermore, various issues were mentioned when measuring the efficiency of periodontal treatments through several economic evaluation techniques (21).

True population-directed actions for preventing periodontal disease are narrow (3), limited data were published on the contribution of oral health services to the prevention and control of periodontal disease (11, 45). Oral health promotion in the form of health education campaigns have been used in the community level using media to obtain sustainable changes in dental health knowledge, attitudes, and behavior (47, 48). However, most of the actions concern dental problems other than periodontal health (3, 49, 50), and only a small number of systematic evaluations of mass education campaigns on periodontal disease have been undertaken (51–56).

Among the strategies that have been described for controlling periodontal disease (5) of special attentions were the population strategy for changing unhealthy lifestyles, particularly those determining tobacco use, alcohol consumption, and oral self-care (plaque removal). The WHO Framework Convention on Tobacco Control (FCTC) was developed as a reaction to the globalization of the tobacco epidemic, smoking being one of the significant risk factors not only for periodontal disease but also for numerous other chronic diseases (3). Regarding the improvement of oral hygiene behaviors, it was suggested that public health interventions should adopt an integrated approach in development of health literacy and appropriate self-care hygiene practices especially in children. Moreover, teaching hygiene skills should be also addressed to caregivers of vulnerable groups in society (e.g., old people, disabled individuals) that may all require special help in maintaining proper oral hygiene (39, 57).

An important role in oral health promotion policy development at a local, national, and international level should be played not only by dental public health practitioners, dental professional organizations, and multi-national commercial companies but also by dental research associations who should improve the evidence base for community actions. Moreover, a collaborative approach should be taken with general public health practitioners and other colleagues from the health, educational, and behavioral sciences sector (39).

The present research topic highlighted several of the abovementioned aspects of the epidemiology (Palma and Leite) and assessment of periodontal disease (Ansai et al.), as well as of the risk factors for this condition, such as tobacco use (Kamath et al.). Moreover, mechanisms involved in the impact of periodontitis on systemic health (e.g., complications in pregnancy) were also discussed (Zi et al.).

Overall, all the articles published under the topic “Periodontal disease – a public health problem” contributed to highlight various aspects of the current state of knowledge of the theoretical framework and raised awareness of the periodontal disease problem as a key part of reorienting health services in order to promote periodontal health.

## Conflict of Interest Statement

The author declares that the research was conducted in the absence of any commercial or financial relationships that could be construed as a potential conflict of interest.
